# Fungal persister cells: The basis for recalcitrant infections?

**DOI:** 10.1371/journal.ppat.1007301

**Published:** 2018-10-18

**Authors:** Jurgen Wuyts, Patrick Van Dijck, Michelle Holtappels

**Affiliations:** 1 VIB-KU Leuven Center for Microbiology, Leuven, Belgium; 2 KU Leuven Laboratory of Molecular Cell Biology, Institute of Botany and Microbiology, Leuven, Belgium; University of Alberta, CANADA

## Abstract

Persister cells are a small subpopulation within fungal biofilms that are highly resistant to high concentrations of antifungals and therefore most likely contribute to the resistance and recalcitrance of biofilm infections. Moreover, this subpopulation is defined as a nongrowing, phenotypic variant of wild-type cells that can survive high doses of antifungals. There are high degrees of heterogeneity and plasticity associated with biofilm formation, resulting in a strong variation in the amount of persister cells. The fraction of these cells in fungal biofilms also appear to be dependent on the type of substrate. The cells can be observed immediately after their adhesion to that substrate, which makes up the initial step of biofilm formation. Thus far, persister cells have primarily been studied in *Candida* spp. These fungi are the fourth most common cause of nosocomial systemic infections in the United States, with *C*. *albicans* being the most prevalent species. Remarkably, persisters exhibit characteristics of a dormant state similar to what is observed in cells deprived of glucose. This dormant state, together with attachment to a substrate, appears to provide the cells with characteristics that help them overcome the challenges with fungicidal drugs such as amphotericin B (AmB). AmB is known to induce apoptosis, and persister cells are able to cope with the increase in reactive oxygen species (ROS) by activating stress response pathways and the accumulation of high amounts of glycogen and trehalose—two known stress-protecting molecules. In this review, we discuss the molecular pathways that are involved in persister cell formation in fungal species and highlight that the eradication of persister cells could lead to a strong reduction of treatment failure in a clinical setting.

## Introduction

**Fig 1:**
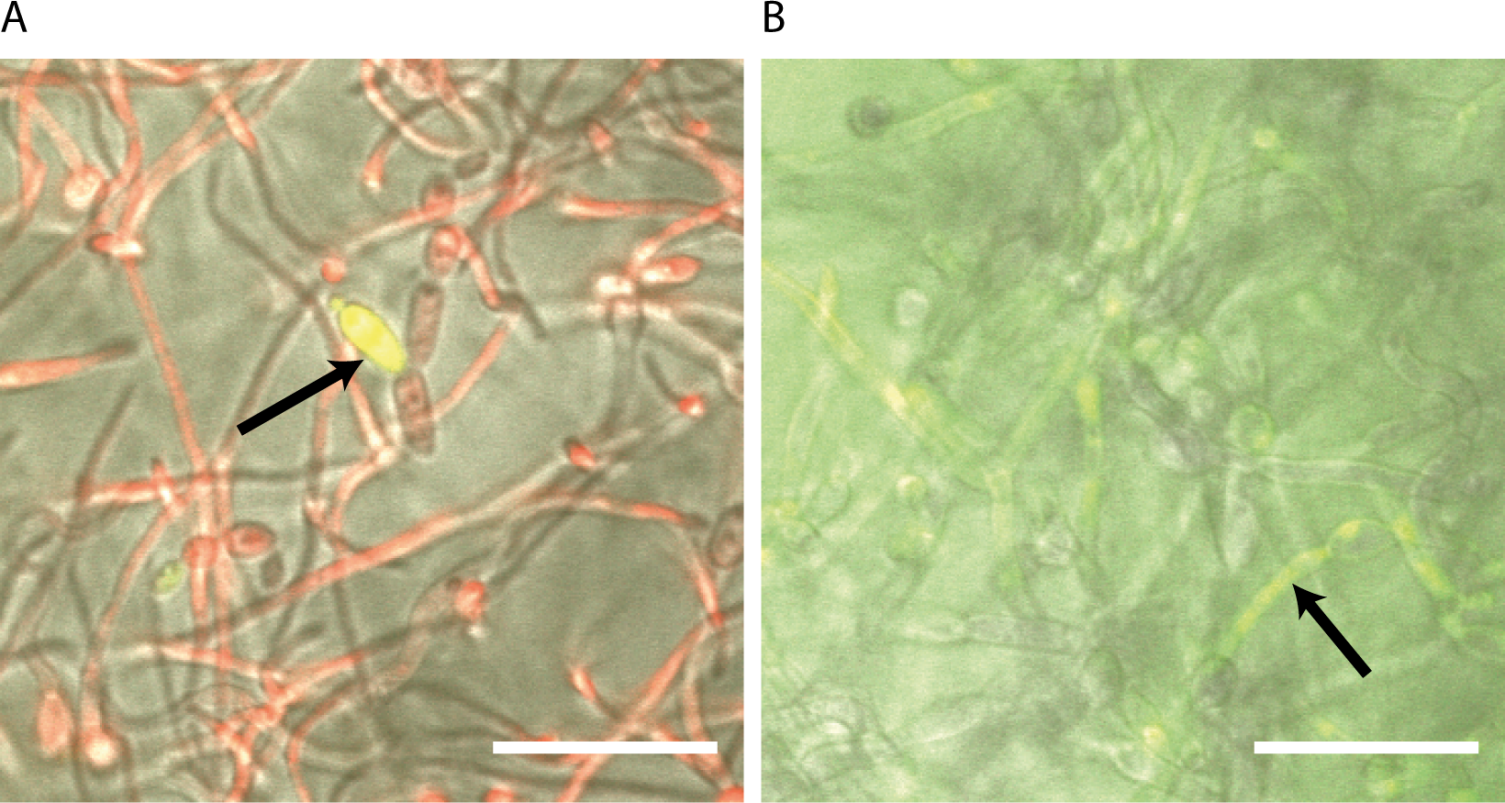
Persister cells are phenotypic variants of wild-type cells. An overnight culture of *C*. *albicans* SC5314 (wild-type) was diluted to OD_600_ 0.1 and seeded to a flat bottomed 96-well plate (CELLSTAR Greiner) containing RPMI-MOPS medium to allow biofilm formation. Biofilms were grown at 37°C for 24 hours, washed with 1× PBS and challenged with 100 μg/mL AmB dissolved in fresh RPMI-MOPS medium or left to mature in fresh RPMI-MOPS medium. After 24 hours, the medium was removed, and the remaining biofilm was again washed with 1× PBS and stained with 100 μg/mL fluorescein diacetate (Sigma Aldrich) and 500 μg/mL Texas Red conjugated to concavalin A (Molecular Probes) for 60 minutes. Fluorescein diacetate stains living cells green, while Texas Red conjugated to concavalin A was used to stain the fungal cell wall red. Pictures were taken using the scanning confocal microscope at a magnification of 600× (white scale bar represents 20 μm). (A) A *C*. *albicans* biofilm treated with AmB lacks green fluorescent cells (indicating that most of the cells are dead). However, a bright fluorescent cell is also present (indicated by the black arrow). This cell meets the definition of a persister cell. We were able to identify several persister cells in the treated biofilm but none of them were of the hyphal form. (B) A nontreated biofilm is thicker in appearance and therefore has a higher background of green fluorescence. Most of the cells in focus are green and thus appear to be viable (indicated by the black arrow). AmB, amphothericin B.

The global AIDS crisis, the use of implants, and the higher survival rates of immunocompromised patients have resulted in an increase in invasive fungal infections [1,2]. *Candida* spp. are the fourth most common cause of bloodstream infections in intensive care units [3] and are associated with mortality rates of up to 40% [4]. Fungicidal compounds currently on the market are able to completely eradicate fast-growing liquid cultures in vitro but are not always successful in clearing fungal infections in a clinical setting [5]. This is extremely problematic, especially in current medical practice in which immunomodulation and device implantation put more patients at risk for fungal infections [6]. Several phenomena can be responsible for treatment failure (e.g., low patient compliance, a lack of antifungal penetration, etc.), but here we will only focus on how pathogens are able to survive fungicidal drug exposure. In this context, we refer to polyenes, such as amphothericin B (AmB), echinocandins, such as caspofungin, and miconazole, a fungicidal azole antifungal drug. Several factors resulting in treatment failure to these drugs were identified [7–9]. First, resistant isolates are not only able to survive high antifungal drug concentrations but are also able to grow in the presence of the fungicidal drug [10]. Second, fungal cells can display tolerance to an antifungal drug. Tolerance is defined as survival following a transient exposure to high concentrations of a fungicidal agent above the minimum inhibitory concentration (MIC) [11]. As a result, it takes longer for a fungicidal agent to kill the cells. Finally, fungal cells can occur as biofilms that are able to attach to biotic surfaces as well as to implantable medical devices [12]. Notably, biofilms are associated with increased resistance against antifungal agents and host immune factors. They can thus result in treatment failure [5]. Several reasons have been proposed for the high resistance of biofilms to antifungal agents, including drug sequestration by matrix components, the up-regulation of drug efflux pumps, and the presence of multidrug-tolerant persister cells [13–15]. Persister cells are a specialized case of tolerance [11]. They are nongrowing, phenotypic variants of wild-type cells and constitute only a small part of the biofilm population that is able to survive high doses of antifungal treatment (Fig 1). When challenged with an increasing amount of a fungicidal drug, they display a biphasic killing pattern by which a large part of the population is killed and a small proportion of the population is able to survive. Moreover, when the cells are regrown and repeatedly challenged with high fungicidal drug concentrations, they display the same biphasic killing pattern [16,17]. An important aspect to take into consideration is that tolerance against fluconazole, often referred to as “trailing growth,” is also observed for fungi [10,18]. However, this is distinct from persister cells. First, persister cells are only observed in biofilms and fluconazole has a limited efficacy against biofilms. Second, fluconazole is a fungistatic agent. Therefore, all cells will survive antifungal treatment, making the distinction of persister cells impossible.

The first evidence for persister cells emerged more than 70 years ago. Joseph Bigger showed that *Staphylococcus aureus* produced metabolic inactive persister cells when treated with penicillin [19]. It took 60 years to confirm that nongrowing persister cells were the cause of this incomplete eradication [20]. We now know that toxin–antitoxin modules are linked to bacterial persister cells. Several different toxin–antitoxin modules have been described, but toxins that inhibit protein translation appear to be the most widespread [21]. Additionally, the activation of bacterial alarmones such as (p)ppGpp have also been shown to promote persister cell development [22]. These intracellular signaling molecules are produced in response to stress. Interestingly, the activation of a stress response in a subpopulation is common in all currently proposed bacterial persister mechanisms [23]. Persister cells in bacteria have been extensively investigated [16,21], whereas less is known about fungal persister cells. Here, we review the mechanisms of *Candida* persister cell development as a contributing factor to therapeutic failure.

### When are *C*. *albicans* persister cells detected?

Persister cells constitute a subset of the biofilm that is refractory to killing, even at very high antifungal drug concentrations. Because of this, they can only be detected when fungicidal drugs are used, but it is not known whether *Candida* persister cells are present in the absence of antifungals. The echinocandins, the polyenes, and one of the azoles (miconazole) share a fungicidal activity against *C*. *albicans* [24–26], but persister cells are typically investigated using the polyene AmB. The development of resistance against AmB is rare [27], which increases the likelihood that the observed surviving colonies are indeed persister cells and not resistant isolates. AmB kills the cells by the sequestration of ergosterol [28]. To achieve this, AmB does not need to enter the cells, and therefore drug efflux pumps are unlikely to be involved in persister cell survival. Although no papers have unambiguously shown that persister cells are present in biofilms treated with echinocandins, the presence of persister cells has been suggested because such treatment also results in a surviving subpopulation [29–31]. The difficulty of interpretation here is that the use of echinocandins is associated with a paradoxical growth effect (PGE), often referred to as “the Eagle effect” [32]. This effect implies that echinocandin activity is attenuated at a certain concentration range above the MIC, caused by the shift from β-glucan, the main target of echinocandins, to chitin synthesis [33]. Because this is observed for a large part of the cell population [34], it is very hard to differentiate from actual persister cells. This implies that the PGE is the result of drug tolerant cells rather than persister cells. Miconazole has been used to investigate the amount of persister cells in *C*. *albicans* biofilms [35], but given the high probability of resistance and the presence of cross-resistance between other azoles (e.g., fluconazole) and miconazole, it is recommended to use AmB instead of miconazole [36]. Second, several emerging non-*albicans* species are intrinsically tolerant against azole antifungals, making comparison of persister cells between species difficult [37,38].

Thus far, *C*. *albicans* persister cells have not been detected in planktonic cultures and therefore appear to be specific to biofilms [14,39,40]. However, biofilm formation itself appears to be dispensable because several mutants defective in biofilm formation produced a normal amount of persister cells [14]. In contrast, adhesion of *C*. *albicans* cells to a surface is critical to observe persister cells [14,40]. *C*. *albicans* persister cells are already present right after adhesion, and although the proportion of persister cells decreases, the total number of persister cells remains constant throughout biofilm development [40]. Persister cell counts are known to be variable, but the variability in the proportion of these cells may be explained, at least partially, by the surface type (Table 1). The wild-type SC5314 strain of *C*. *albicans* did not produce any persister cells when grown on polyvinylchloride (PVC) in YNB-MOPS medium, whereas two other studies did observe persister cells for this strain. Both used polystyrene as a substrate, and the strains were grown in RPMI-MOPS or YNB medium [39–41]. Therefore, the surface type appears to mediate the proportion of persister cells. A protocol was developed to critically assess the fraction of persister cells and to standardize persister cell research [17].

**Table 1:** Overview of the occurrence of persister cells in *Candida* spp. depending on growth parameters.

Species	Strain	Antifungal	Proportion of persister cells	Substrate	Medium	Culture conditions before challenge	Reference
*C*. *albicans*	3153A	100 μg/mL AmB	1%–2%	Flat-bottomed microtiter plate (polystyrene)	RPMI-MOPS	48 h, 37°C 100 RPM	[14]
		100 μg/mL chlorohexidin	1%–2%				
	CAI4	100 μg/mL AmB	0.05%–0.1%				
	SC5314		Not detected	PVC discs	YNB-MOPS (50 mM glucose)	48 h, 37°C	[39]
	GDH 2346		0.01%				
*C*. *krusei*	Glasgow strain		0.001%				
*C*. *parapsilosis*	AAHB 4479		0.07%				
*C*. *glabrata*	AAHB12		Not detected				
*C*. *tropicalis*	AAHB73						
*C*. *albicans*	CA-IF100	2.4 mM miconazole	1.3%–2.3%	?^a^	RPMI-MOPS	16 h	[35]
	SC5314	100 μg/mL AmB	0.012%	Flat-bottomed microtiter plate (polystyrene)		24 h, 37°C, 100 RPM	[40]
	3153A		0.014%				
	YEM30		0.012%				
*C*. *albicans*	SC5314	256 μg/mL AmB	0.46%	24-well plate (polystyrene)	YNB (100 mM glucose)	48 h, 37°C, 80 RPM	[41]
	BF-1		0.01%				
*C*. *glabrata*	ATCC90030		0.03%				
	T1570		Not detected				
*C*. *tropicalis*	ATCC13803		0.17%				
	T1427		0.07%				
*C*. *albicans*	GZY803	32 μg/mL AmB	0.1%	Flat-bottomed microtiter plate (polystyrene)	RPMI-MOPS	72 h, 37°C	[42]
	BWP17		1%				
*Saccharomyces cerevisiae*	Σ 1278b YS-11	100 μg/mL AmB	0.4%	microtiterplate (polystyrene)	YPD	48 h, 30°C	[43]

^a^Not specified

**Abbreviations:** AmB, amphothericin B; PVC, polyvinylchloride; RPM, revolutions per minute.

### Nutrient sensing and metabolic control coordinate *C*. *albicans* persister development

**Fig 2:**
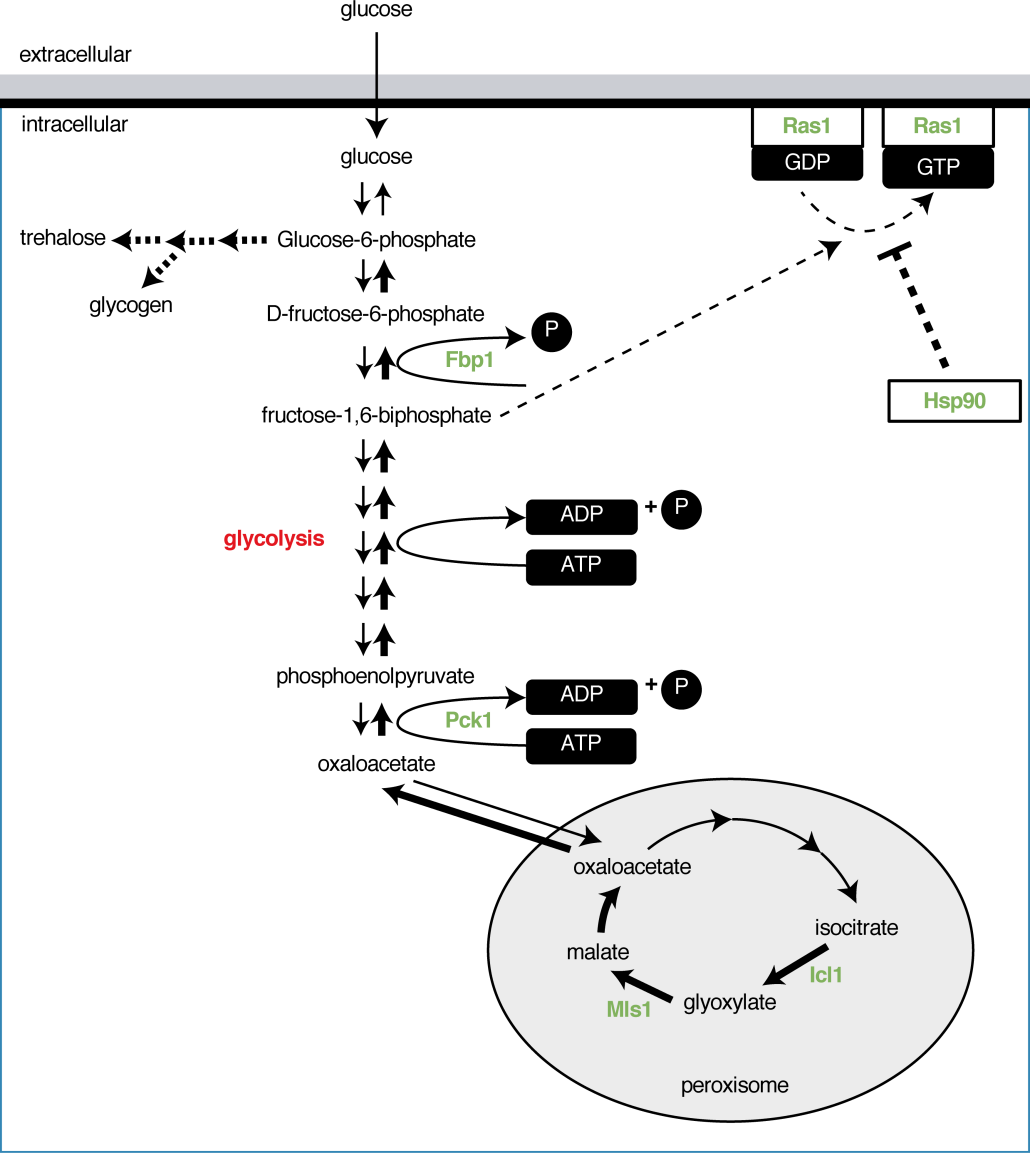
Persister cells of *C*. *albicans* are committed to energy storage. Several enzymes of energy storage pathways are up-regulated in persister cells (indicated in green), whereas glycolysis is down-regulated (indicated in red), suggesting that *C*. *albicans* persister cells are adapted to the absence of glucose. Icl1 and Mls1 are up-regulated in persister cells. This results in an increase in oxaloacetate that enters gluconeogenesis that is converted to phophoenolpyruvate by Pck1. Pck1, together with Fbp1, are also up-regulated in persister cells and are both key enzymes in the gluconeogenesis pathway. The up-regulation of these enzymes will likely result in an increased flux towards the production of energy storage molecules such as trehalose and glycogen. Additionally, up-regulation of Fbp1 also results in a decreased amount of fructose-1,6-biphosphate, which is the activator of Ras1. This suggests that persister cells have a lower proportion of active Ras1. Finally, Hsp90, which is up-regulated in persister cells may also inhibit the activation of Ras1 in persister cells. Ras1 is up-regulated in persister cells, and because persister cells appear to have a lower proportion of active Ras1, this may prepare persister cells for a rapid metabolic switch to restart proliferation. Dashed bold lines: likely to occur in persister cells; bold lines: proven in persister cells; dashed lines: likely to occur in cells growing in the presence of glucose. All other lines: proven to occur in cells growing in the presence of glucose. Fbp1, fructose-1,6-biphosphatase; GDP, guanosine-5'-diphosphate; GTP, guanosine-5'-triphosphate; Hsp90, heat shock protein 90;Icl1, Isocitrate lysase; Mls1, malate synthase; Pck1, phosphoenolpyruvate carboxykinase; Ras1, rat sarcoma.

Glucose is the preferred carbon source for *C*. *albicans*. In the absence of glucose, fungal cells are forced to use alternative carbon sources. The proteome signature of persister cells suggests that they survive on such alternative carbon sources (Fig 2). First, two enzymes, isocitrate lyase (Icl1) and malate synthase (Mls1), which are located in the peroxisomes [44], are up-regulated [41]. Up-regulation of this pathway, which is down-regulated in the presence of glucose, bypasses the decarboxylation steps of the tricarboxylic acid (TCA) cycle to produce gluconeogenesis precursors [45,46]. Second, fructose-1,6-biphosphatase (Fbp1) and phosphoenolpyruvate carboxykinase (Pck1) are up-regulated in *C*. *albicans* persister cells [41]. Pck1 is also down-regulated in the presence of glucose [45], again suggesting that persister cells survive on alternative carbon sources. Additionally, Fbp1 is the rate-limiting step for gluconeogenesis and importantly, Fbp1 converts fructose-1,6-biphosphate to D-fructose-6-phosphate, thereby decreasing the concentration of fructose-1,6-biphosphate. It is known that in *Saccharomyces cerevisiae*, fructose-1,6-biphosphate activates Ras1 [47]. The up-regulation of Fbp1 and the concomitant lower amount of fructose-1,6-biphosphate may result in a lower amount of active Ras1 in persister cells. Additionally, the higher heat shock protein 90 (Hsp90) levels in persister cells may also keep Ras1, which is present in higher concentrations in persister cells, in the inactive form [41,48,49]. A higher amount of inactive Ras1 may prepare the cells for a rapid metabolic switch after glucose addition to restart proliferation. Finally, all major glucose utilization pathways, such as the tricaboxylic acid cycle, glycolysis, and pentose phosphate pathway, are down-regulated in persister cells [41]. Downregulation of glycolytic proteins in persister cells suggests that they have low levels of ty1-mediated expression (Tye7) [50], resulting in cellular commitment to energy storage.

Glucose starvation results in a dormant state, as is observed for persister cells. However, it is important to note that this is similar for glucose-starved cells in planktonic cultures, but no persister cells are observed there [14,39,40]. This indicates that surface adhesion primes part of the cell population for survival upon antifungal treatment. Unfortunately, the molecular switch responsible for this phenomenon remains unknown.

### Increased oxidative stress responses mediate fungal persister cell survival

**Fig 3:**
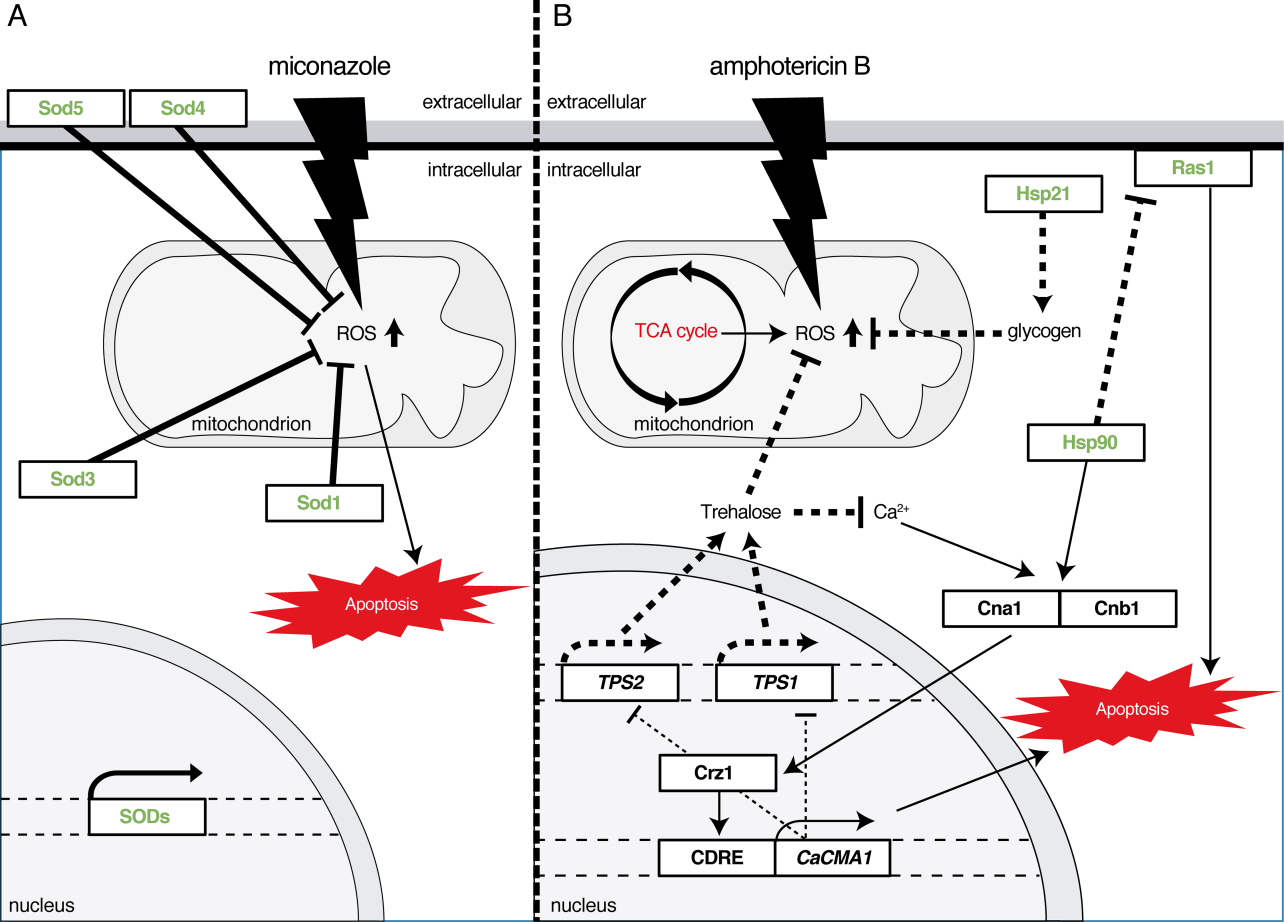
Persister cells are able to survive ROS accumulation induced by fungicidal drugs. Fungicidal drugs induce ROS accumulation in the mitochondria, and persister cells appear to use several mechanisms to cope with the high amount of ROS. (A) Miconazole-induced ROS in the mitochondria is detoxified by the overexpression of several SODs. (B) AmB treatment results in ROS accumulation. This leads to calcium accumulation and activation of the calcineurin pathway, thereby increasing expression of *CaCMA1* via Crz1. *CaCMA1* expression results in decreased expression of *TPS1* and *TPS2* and concomitantly results in apoptosis. In order to survive the apoptotic response induced by ROS, persister cells need to break the apoptotic feedback loop. They do so by up-regulation of stress response proteins (indicated in green). First, up-regulation of Hsp21 in persister cells may result in an increased glycogen content that protects the cells from the oxidative stress. Second, Hsp90 is also up-regulated in persister cells. Paradoxically, Hsp90 also activates the apoptotic pathway by activation of the calcineurin pathway, and concomitantly inhibits Ras1-cAMP-PKA signaling, decreasing the apoptotic response. Therefore, depending on the conditions, Hsp90 may determine the cellular fate of biofilm cells under stringent oxidative stress conditions and may direct the cells to apoptosis or the persister cell state. Dashed bold lines: likely to occur in persister cells; bold lines: proven in persister cells; all other lines: proven to occur in cells undergoing apoptosis. AmB, Amphotericin B; Crz1, calcineurin responsive zinc finger 1; Hsp21, heat shock protein 21; PKA, protein kinase A; Ras1, rat sarcoma; ROS, reactive oxygen species; SOD, superoxide dismutase; Tps1, trehalose-6-phosphate synthase.

Fungicidal drugs have been shown to induce apoptosis-like cell death in fungi [51–53]. This coincides with the production of reactive oxygen species (ROS) and metacaspase activation [54] (Fig 3). In contrast, persister cells are able to survive high concentrations of antifungals known to induce ROS accumulation [41]. Therefore, to survive, they need to activate the oxidative stress response and must have mechanisms to prevent them from going into apoptosis (Fig 3). This has been demonstrated by several studies. First, persister cells have an increased expression of superoxide dismutases (SODs), which provide these cells with protection against miconazole-induced ROS production (Fig 3A) [35]. To show the importance of this overexpression of SODs in persister cells, experiments were performed in which the SOD inhibitor, N,N’-diethyldithiocarbamate (DDC), was added together with miconazole, showing a strong reduction in the proportion of persisters. Further support was shown by experiments using the homozygous *SOD4 SOD5* double deletion strain, which resulted in a lower frequency of persister cells, in the presence of miconazole. However, both the double deletion mutant and the use of a SOD inhibitor failed to completely eradicate fungal persister cells, indicating that other SODs or additional mechanisms are also important. Second, a small heat-shock factor, Hsp21, is upregulated in persister cells and is important in ROS protection (Fig 3B) [41]. This was demonstrated when menadione-induced ROS led to a severe growth defect in a *HSP21* deletion mutant [55]. This mutant had lower levels of glycogen, a molecule with a crucial role in cellular protection under stressful conditions. Complementation of the mutant resulted again in higher glycogen levels and protection against ROS. Third, expression of alkyl hydroperoxide reductase 1 (*AHP1)* correlates with persister levels in the presence of high concentrations of AmB [42,56] and is important in the adaptive response to oxidative stress [57]. This was demonstrated by comparing the susceptibility profile of a diploid strain (BWP17) and a haploid strain (GZY803) for increasing AmB concentrations. The haploid GZY803 strain had a reduced susceptibility indicated by a lower amount of persister cells and a lower minimum biofilm inhibitory concentration (MBIC). Overexpression of *AHP1* in the haploid strain resulted in an increase in the persister cell population, while the MBIC remained unaffected. Because they used a tetracycline-inducible promoter (Tet-off) for overexpression, they could demonstrate that upon the addition of doxycycline (20 μg/mL), the persister fraction was reduced again to the original level. Although this demonstrates that *AHP1* is important in the fraction of persister cells, it is important to note that only 32 μg/mL of AmB was used compared to the usual 100 μg/mL in other studies. Finally, histone deacetylases (HDAs) appear to be important to inhibit apoptosis. This was shown by the addition of HDA inhibitors, of which some resulted in complete biofilm eradication when added together with AmB, even at relatively low AmB drug concentrations [53]. It remains unclear how these HDA inhibitors are able to kill persister cells, but the addition of valproate—which is one of the HDA inhibitors—is known to induce apoptosis and requires yeast caspase 1 (Yca1) for this in *S*. *cerevisiae* [58]. This suggests that persister cells must have the capacity to inhibit the activity of Yca1. The Yca1 orthologue of *C*. *albicans*, metacaspase 1 (Mca1), is also involved in apoptosis as it is required for calcineurin-induced apoptosis, which partially depends on Hsp90 (Fig 3B) [59]. Strains with a compromised *HSP90* expression showed less apoptosis, because the calcineurin-caspase pathway was downregulated in these strains [59]. However, as noted above, Hsp90 is upregulated in persister cells [34] and remarkably, Hsp90 is also known to inhibit Ras1 [55]. A decrease in activation of the Ras1-cAMP-protein kinase A (PKA) pathway results in a supressed apoptotic response [60], indicating that Hsp90 may determine the cellular fate of biofilm cells. Therefore, the exact relationship between Hsp90, persister cells, and apoptosis remains to be determined.

In addition to promoting apoptosis directly, Mca1 also promotes apoptosis indirectly. The deletion of *MCA1* resulted in a high accumulation of trehalose through the increased transcription of *TPS1* and *TPS2*, two genes directly related to trehalose synthesis [61]. Trehalose is another stress protectant and also protects the cells by inhibiting calcineurin-induced apoptosis, which is dependent on the metacaspase Mca1 [62]. This suggests that persister cells accumulate a high amount of stress protectants to survive ROS-induced apoptosis. Stress protectants such as glycogen and trehalose protect the cells by stabilizing proteins and thereby act as chemical chaperones.

### Do persister cells contribute to in vivo persistence?

MICs assays have been developed to predict the susceptibility of clinical isolates to antifungals and are employed by clinical microbiology laboratories to predict treatment outcome. This is successful for some antifungals, but not for AmB, indicating that other phenomena are important [63]. Indeed, chronic oral thrush in cancer patients receiving a daily 0.2% chlorohexidine solution harboured high persister (*hip*) strains when oral carriage of *C*. *albicans* was longer than 8 weeks [64]. The isolated *hip* strains developed persister cells at frequencies of up to 9%. It is important to note that some, but not all, also had increased MICs. Patients with only transient oral carriage of *C*. *albicans* (<8 weeks) did not have an increased proportion of persister cells compared to lab strains. Although this indicates that oral carriage may select for *hip* strains, it is not known whether these strains also contribute to recalcitrant infections because they were isolated from asymptomatic patients.

Upon infection of the human host, fungi are attacked by phagocytes such as neutrophils and macrophages [65]. Phagocytes are able to recognize cell wall components, and this results in phagocytosis. Fungal persister cells have up-regulated cell wall integrity pathways to cope with the membrane stress exerted by AmB [41]. This suggests that persister cells can have a different cell wall composition. This may result in different phagocytosis and concomitantly ROS production of the phagocytes [65]. Once inside the phagocytes, the persister cells face a nutrient-limited and stressful environment [21]. To survive phagocytosis, fungal cells need a functional glyoxylate pathway and enhanced oxidative stress response [66]. Both are up-regulated or important for persister cell formation in vitro [35,41]. However, these pathways were investigated in vitro. Therefore, it is unclear whether they also contribute to immune evasion and relapsing infections in vivo.

An increased extracellular matrix (ECM) production was suggested to occur in persister cells because a glucanosyltransferase (Bgl2) and a exoglucanase (Xog1) are upregulated [41]. Both enzymes are essential for ECM production, because the deletion of their corresponding genes results in biofilms with a decreased ECM [67]. Moreover, *KRE1* and *SKN1* are also involved in ECM production and are overexpressed in persister cells [15]. Because persisters only constitute 1% of the biofilm, additional ECM produced by persister cells should provide extra protection to all the cells in the biofilm as ECM is then a public good. It was previously shown that non–matrix-producing cells in a bacterial biofilm benefit from those that produce ECM [68,69]. The ECM of *C*. *albicans* is associated with increased tolerance against antifungals [67,70] and inhibits the formation of neutrophil extracellular traps (NETs). These are web-like structures composed of histones, DNA, and proteins that mediate the killing of fungal cells in vivo [71]. Although *KRE1* and *SKN1* deletion were associated with increased NET activation, it is unclear whether Xog1 and Bgl2 are also important because they were not included in a screen for mutants with enhanced NET formation [71]. In summary, persister cells appear to produce more ECM, thereby contributing to the protection of the biofilm in vivo by inhibiting NET formation.

Several virulence factors also contribute to the infection potential of persister cells. As noted earlier, Hsp90 is up-regulated in persister cells [41]. This heat-shock factor is vital for the dispersal of *C*. *albicans* biofilms [72], whereas concomitantly, other virulence factors are also up-regulated in persister cells; the invasins stress-seventy subfamily A 1 (Ssa1) and agglutinin-like sequence 3 (Als3) [41] are crucial for tissue adherence and host invasion [73]. More importantly, a small fraction of cells able to survive antifungal treatment may facilitate the development of antifungal drug resistance. Interestingly, Hsp90 is also involved in the development of resistance. First, AmB resistance requires high levels of Hsp90 to allow the cells to cope with diverse stresses caused by mutations that confer AmB resistance [27]. Second, Hsp90 governs antifungal drug resistance to azoles and echinocandins, and this is dependent on calcineurin [74,75]. Therefore, persister cells appear to promote resistance development.

### Beyond *C*. *albicans* persister cells

Thus far, this review has focused on *C*. *albicans*. However, other *Candida* spp. are also known to develop persister cells. *C*. *glabrata* does not appear to produce a high proportion of persister cells. Only one out of three strains produced persister cells, albeit at lower fractions (Table 1) [39,41]. Thus far, *C*. *glabrata* persister cells have only been investigated using YNB medium, and the use of this medium appears to result in a low fraction of persister cells. *C*. *tropicalis*, *C*. *krusei*, and *C*. *parapsilosis* have all been shown to produce persister cells [39,41]. However, for *C*. *tropicalis* (and *C*. *albicans*) this appears to be media-, strain-, and/or substrate-dependent because one study was unable to observe any persister cells [39]. Similar to *C*. *albicans*, thus far no persister cells have been detected in planktonic cultures in *C*. *glabrata*, *C*. *tropicalis*, *C*. *krusei*, or *C*. *parapsilosis*, indicating that *Candida* persister cells are growth-phase specific [14,39,40].

The *S*. *cerevisiae* Σ1278b YS-11 strain also produces persister cells. However, this tolerant subpopulation appears to develop independently of the growth mode [76]. Bojsen and colleagues investigated *S*. *cerevisiae* persister cells when challenged with high concentrations of AmB (up to 100 μg/mL) for a period of 24 hours and demonstrated that *S*. *cerevisiae* persister cell survival mechanisms, such as those for *C*. *albicans* [14], are not heritable. Second, they discovered that planktonic cultures of *S*. *cerevisiae* also produce drug-tolerant persister cells. However, only a lower concentration of AmB was used (10 μg/mL), instead of the usual high concentrations (>100 μg/mL), and the cells were only challenged for 12 hours. It is not known whether these persister cells would still survive more stringent conditions, so these cells may not be “true” persister cells. Finally, the responsible pathways for *S*. *cerevisiae* persister cell survival in both planktonic and biofilm cultures were investigated using barcode sequencing of pooled mutants. Notably, they could link both the target of rapamycin C (TORC) and Ras pathway to a high persister phenotype, independent of the growth mode.

### Outlook and perspectives

Many questions about *Candida* persister cells remain, but single-cell technologies and technologies to study antifungal dynamics will undoubtedly contribute to understanding how a small subpopulation of biofilm cells can develop differently, despite the fact that they are growing in similar environmental conditions. These technologies may also elucidate how this small subpopulation may have such huge consequences for a patient. This will lead to insights into biofilm plasticity and on how persister cells contribute to recalcitrant infections. Bacterial persister researchers have already employed microfluidics tools to identify and isolate persister cells by analyzing the population of cells that survive antibiotic treatment and that start to grow again upon removal of the antibiotic [20]. Similar strategies could be employed for fungal perister cells. This would ultimately pave the way for new strategies to develop antifungals. Although little is known about fungal persister cells, several interesting targets have emerged. Hsp21 appears to be important in persister cell formation and is absent in humans, making it an ideal target for antifungal drug development. Thus far, no treatment options exist to completely eradicate fungal biofilms, but bacterial persister cells can be eradicated in vivo [77], providing hope that one day, fungal biofilms may be eradicated completely.
